# A Three-Dimensional Comparison of Tick-Borne Flavivirus Infection in Mammalian and Tick Cell Lines

**DOI:** 10.1371/journal.pone.0047912

**Published:** 2012-10-24

**Authors:** Danielle K. Offerdahl, David W. Dorward, Bryan T. Hansen, Marshall E. Bloom

**Affiliations:** 1 Laboratory of Virology, Rocky Mountain Laboratories, NIAID, NIH, Hamilton, Montana, United States of America; 2 Microscopy Unit, Research Technology Branch, Rocky Mountain Laboratories, NIAID, NIH, Hamilton, Montana, United States of America; Kansas State University, United States of America

## Abstract

Tick-borne flaviviruses (TBFV) are sustained in nature through cycling between mammalian and tick hosts. In this study, we used African green monkey kidney cells (Vero) and *Ixodes scapularis* tick cells (ISE6) to compare virus-induced changes in mammalian and arthropod cells. Using confocal microscopy, transmission electron microscopy (TEM), and electron tomography (ET), we examined viral protein distribution and the ultrastructural changes that occur during TBFV infection. Within host cells, flaviviruses cause complex rearrangement of cellular membranes for the purpose of virus replication. Virus infection was accompanied by a marked expansion in endoplasmic reticulum (ER) staining and markers for TBFV replication were localized mainly to the ER in both cell lines. TEM of Vero cells showed membrane-bound vesicles enclosed in a network of dilated, anastomosing ER cisternae. Virions were seen within the ER and were sometimes in paracrystalline arrays. Tubular structures or elongated vesicles were occasionally noted. In acutely and persistently infected ISE6 cells, membrane proliferation and vesicles were also noted; however, the extent of membrane expansion and the abundance of vesicles were lower and no viral particles were observed. Tubular profiles were far more prevalent in persistently infected ISE6 cells than in acutely infected cells. By ET, tubular profiles, in persistently infected tick cells, had a cross-sectional diameter of 60–100 nm, reached up to 800 nm in length, were closed at the ends, and were often arranged in fascicle-like bundles, shrouded with ER membrane. Our experiments provide analysis of viral protein localization within the context of both mammalian and arthropod cell lines as well as both acute and persistent arthropod cell infection. Additionally, we show for the first time 3D flavivirus infection in a vector cell line and the first ET of persistent flavivirus infection.

## Introduction

Vector-borne flaviviruses, such as Dengue (DENV), Yellow Fever, Japanese Encephalitis virus (JEV), and tick-borne encephalitis (TBEV) viruses are recognized as significant human pathogens and cause considerable mortality and morbidity worldwide. TBEV, a tick-borne flavivirus (TBFV), is responsible for 14,000 infections per year [1] and has a fatality rate of up to 40% [2]. Symptoms of TBEV infection can include fever, malaise, meningitis, and encephalitis. TBEV and other TBFV, such as Omsk Hemorrhagic Fever virus, are classified as NIAID Category C pathogens and are treated as biosafety level 4 agents in the United States. One TBFV, Langat virus (LGTV), is naturally attenuated [3], [4], making it suitable for biosafety level 2 work and ideal for use in laboratory studies as a model for higher pathogenicity viruses.

In nature, LGTV and other TBFV maintain a complex cycle between ticks and vertebrate hosts. Historically, Ixodidae ticks (hard ticks) have been considered to be the arthropod vector, but, some findings with Alkhurma virus [5] and Kyasanur Forest virus [6] suggest that the soft-bodied *Ornithodoros* ticks can transmit TBFV. Thus, the arthropod host-range for TBFV may be greater than assumed. The TBFV present a unique situation because the viruses persistently infect ticks and are maintained by vertical transmission across the developmental instars (larval, nymph, and adult). Horizontal transmission (from tick to vertebrate host) then allows amplification of the frequency of the virus within the tick population, as uninfected ticks feeding upon infected vertebrates can acquire the virus [7], [8]. The primary vertebrate hosts are generally small rodents; however, infection of larger mammals also occurs in endemic areas. Humans are an inadvertent, dead-end host, contracting TBFV via tick bite or less frequently by consumption of milk from infected animals [1]. The impact of TBFV infection on vertebrates can vary considerably; some reports describe persistent infection of vertebrates and vertebrate cell lines [9]–[11] while other laboratory studies show severe disease development in infected animals [12]–[14].

Like other members of the family *Flaviviridae,* LGTV is a single-stranded, positive-sense RNA virus. Upon infecting a cell, the virions are thought to traffic to the endosome, where they undergo structural transformation and fuse with host cell membranes, releasing the 11-kb viral RNA genome into the cytoplasm [15]. The genomic RNA, which can function as mRNA, is translated into an approximately 400 kDa polyprotein [16] that is subsequently cleaved into at least ten proteins by both viral and cellular host proteases [17]. The currently defined complement consists of three structural proteins (Capsid [C], membrane [M], and envelope [E]) and seven nonstructural proteins (NS1, NS2A, NS2B, NS3, NS4A, NS4B, and NS5) [17], [18]. Precise functions for all of the nonstructural proteins are not fully elucidated, but some roles have been determined. NS1 can be found on the cell surface but is known to be secreted [17] and is also detected in perinuclear areas where it is believed to play a part in viral replication [19]; however, its role has not yet been clearly defined. NS3 is a RNA helicase and also acts as a viral protease using NS2B as a co-factor [20], [21]. NS4A, a small hydrophobic protein, has been implicated in the cellular changes that accompany virus reproduction [22] and has been shown to localize to foci within the perinuclear region of infected cells [23]. NS5 is the RNA-dependent RNA polymerase and methyltransferase [17]; however, recent implications of LGTV NS5 as an innate immune response antagonist confirms a broader role for this protein beyond genome replication [24]. This broader role is also seen in NS5′s wider distribution, being located in cell nuclei, perinuclear regions, and diffusely throughout the cytoplasm [25]. Following synthesis of the viral proteins, the single stranded genome is converted into a double-stranded RNA (dsRNA), which serves as a transcriptional template as well as the viral replicative form. Together with the replication complex, made up of NS1, NS2A, NS3, NS4A, and NS5, dsRNA is found in cytoplasmic, perinuclear foci throughout LGTV infected cells. This dsRNA species is an additional obligatory replicative intermediate.

Dramatic cellular changes accompany flavivirus replication and have been the subject of considerable study, although most of the studies have been done in mammalian cells using the mosquito-borne flaviviruses. Replication has been linked to areas of the cell containing large amounts of virus-induced structures, including convoluted membranes, dilated cisternae, vesicles, tubules, and paracrystalline arrays [19], [26]–[28]. These structures are localized in a perinuclear distribution of vastly proliferated membranes which, in the case of cells infected with mosquito-borne flaviviruses, are derived from endoplasmic reticulum (ER) [29]–[31]. The vesicles are typically 70–150 nm in diameter [28] with a pore-like connection either between vesicles and the cytoplasm or between individual vesicles [30], [31].

Vector-borne flaviviruses, like DENV, JEV, and TBEV, must replicate in both mammalian and arthropod cells, and a limited number of comparative studies have been published [32]–[36]. The findings, generally, show agreement between mammalian and arthropod cells in virus induced structures, such as cytoplasmic membrane proliferation and vesicle formation. Some changes were noted via TEM by Senigl *et al*. [35] four to seven days post TBEV infection; viral particles were observed inside rough ER in mammalian cells but were visualized within vacuoles or within the cytosol in tick cells. Consequently, we have undertaken a direct comparison of LGTV infection in both mammalian and arthropod cells, and extended the results to include 3D reconstructions. In addition, we have shown for the first time the 3D structure of acute and persistent flavivirus infection in an arthropod cell line.

## Materials and Methods

### Cells and Viruses

African green monkey kidney cells (Vero, ATCC) were maintained in Dulbecco’s minimal essential media (DMEM, Invitrogen) supplemented with 10% fetal calf serum (FCS) and 50 ug/ml gentamicin (complete DMEM) at 37°C in 5% CO_2_. ISE6 cells derived from *I. scapularis* embryonated eggs (kindly provided by Dr. Timothy Kurtti, University of Minnesota) were cultured at 32°C as previously described [37] with the addition of 10 ug/ml gentamicin to the L-15B media.

A virus stock of Langat virus strain TP21 (LGTV) (kindly provided by Dr. Alexander Pletnev, NIH/NIAID) was prepared by infection of Vero cell cultures at a multiplicity of infection (MOI) of 0.005 [38], [39]. For virus titration by immunofocus assay, 1×10^5^ Vero cells were seeded in complete DMEM into wells of twelve-well plates. Ten-fold dilutions of LGTV were adsorbed for 60 minutes at 37°C, 5% CO_2_ with constant rocking. Infected cells were then washed twice with Dulbecco’s phosphate buffered saline (DPBS, Invitrogen) and fresh complete DMEM added. After three days incubation, cells were washed twice with DPBS and fixed with 100% methanol for 30 minutes. Cells were rinsed with DPBS, blocked with OptiMEM supplemented with 2% FCS for 10 minutes, and incubated with Russian Spring-Summer Encephalitis immune ascites fluid (ATCC) diluted 1∶1000 in OptiMEM/2% FCS. Following two DPBS washes, cells were incubated with 1∶1000 dilution of anti-mouse horseradish peroxidase antibody (Dako) in OptiMEM/2% FCS. Foci were visualized using peroxidase substrate containing 0.4 mg/ml 3, 3′ diaminobenzidine (Sigma Aldrich) and 0.0135% H_2_O_2_ (JT Baker) in PBS. For all experiments, Vero and ISE6 cells were infected at an MOI of 10.

### Persistent Infection of ISE6 Cells

To establish a persistently infected culture, 1×10^7^ ISE6 cells were plated in a 150 cm^2^ flask and incubated for 24 hours at 32°C. Cells were then infected at an MOI of 10 with LGTV TP21. Cells were passaged every two to three weeks as needed and supernatants collected for viral titer via immunofocus assay to confirm productive viral infection. Samples for immunofluorescence and electron microscopy were prepared at multiple time points post-infection. No differences were noted between samples collected at various time points. Representative images for figures depicted were acquired between 90 and 260 days post infection.

### Antibodies

The primary antibodies against viral proteins used were: mouse monoclonal anti-prM 13A10, IgG2a; mouse monoclonal anti-E 11H12, IgG2a; mouse monoclonal anti-NS1 6E11, IgG2a (all kind gifts from Dr. Connie Schmaljohn, USAMRID, Fort Detrick, Frederick, MD), chicken poly-clonal anti-NS3 (sequence: CZRDIREFVSYASGRR) and chicken polyclonal anti-NS5 (sequence: CZDRHDLHWELKLESSIF) (custom prepared by Aves Labs), mouse anti-dsRNA clone J2 (purchased from English & Scientific Consulting, Szirak, Hungary). Markers against cellular organelles used were: Alexa Fluor 594-conjugated wheat germ agglutinin (WGA, Invitrogen), and mouse monoclonal Dylight 488-conjugated protein disulfide isomerase 1D3 (PDI, Enzo Life Sciences). Secondary antibodies used were: Alexa Fluor 488- and 594- conjugated anti-mouse- and anti-rabbit-specific IgG and Alexa Fluor 647-conjugated anti-chicken-specific IgG (Invitrogen).

### Immunofluorescence Microscopy

Vero and ISE6 cells were seeded at 3×10^4^ cells/well in 8 well Labtek dishes (Nunc), infected with LGTV (MOI 10), and incubated for 48 hours at 37°C and 32°C, respectively. The cells were washed twice with PBS, fixed for 10 minutes in 4% paraformaldehyde (PFA)/5% sucrose in PBS, permeabilized with 0.1% Triton X-100/4% PFA for 10 minutes, washed with 50 mM glycine for 5 minutes, and blocked for 60 minutes with 2% bovine serum albumin (BSA)/PBS. Primary and secondary antibodies were incubated at a 1∶1000 dilution in 2% BSA/PBS for 60 minutes each, with three five-minute washes with PBS between incubation steps. After washes, slides were dried and coverslips mounted using Prolong Gold Antifade with DAPI (4′, 6-diamidino-2-phenylindole, Invitrogen). Confocal images were acquired using a Carl Zeiss LSM 710 confocal laser scanning microscope. TIFF files of individual channels and merge images were made using Bitplane Imaris 7.2 and images assembled for publication using Adobe Photoshop software.

### Electron Microscopy Processing

For transmission electron microscopy (TEM), Vero or ISE6 cells were seeded on 13-mm Thermanox coverslips (Nunc) in 12 well plates at 1×10^5^ cells/well. After 24 hours, cells were infected with LGTV TP21 at an MOI of 10 and incubated for 48 hours at 37°C and 32°C, respectively. Cells were then washed twice with DPBS and fixed with 2.5% glutaraldehyde in cacodylate buffer (100 mM sodium cacodylate, 50 mM KCl, 2.5 mM CaCl_2_) for 30 minutes at room temperature prior to overnight incubation at 4°C.

Samples were processed further in a Biowave model laboratory microwave oven, equipped with a Coldspot water circulator (Ted Pella, Inc.) as follows: washed twice in 0.1 M sodium phosphate buffer, pH 7.2, for 1 minute each; post-fixed in 1% osmium tetroxide in phosphate buffer for two cycles of 2 minutes on, 2 minutes off, and 2 minutes on; washed once in phosphate buffer for 1 minute and twice in water for 1 minute each; contrasted with 1% uranyl acetate in water for two cycles of 2 minutes on, 2 minutes off, and 2 minutes on; dehydrated in three changes of ethanol for 1 minutes each; and embedded in Spurr’s resin using steps of 50%, 75%, and two changes of 100% resin in ethanol for two periods of 5 minutes each. The power output of the oven was set at 250 watts for dehydration and embedding. All other steps were performed at a setting of 167 watts. The cover slips were placed cell-side down onto resin block molds, polymerized overnight at 65°C, and removed from hardened blocks after a 5 second immersion in liquid nitrogen.

Thin and semi-thick sections of approximately 70 and 250 nm, respectively, were cut using a diamond knife and a model EM UC6 microtome (Leica Microsystems). Thin sections were collected on uncoated 200 mesh copper grids. Once thoroughly dried, the sections were stained with fresh 1% lead citrate using 1 minute of microwave irradiation at 167 watts, followed by a 1 minute water wash at 167 watts and standard drop-wise washing.

For electron tomography (ET), semi-thick sections were collected on carbon coated 200 mesh copper grids (Ted Pella, Inc.), dried, and stained and washed similarly, except that the microwave irradiation steps were extended to 2 minutes each. Tomography grids were then immersed briefly in goat anti-mouse IgG 10 nm gold conjugate (Ted Pella, Inc.) to add fiducial markers on both sides of the sections and then thoroughly washed with water.

### Electron Microscopy Imaging and Tomography

Thin sections were examined at 80 kV using a model H7500 transmission electron microscope (Hitachi High Technologies, Inc.). Digital images were captured using a model HR-100 camera system (Advanced Microscopy Techniques, Inc.) and processed with PhotoShop (Adobe Systems, Inc.). Semi-thick sections were examined at 120 kV using a G2 Spirit BioTwin model TEM (FEI, Inc.) equipped with a tilt stage, Xplore3D acquisition system, and model Ultrascan 1000 CCD system (Gatan, Inc.). Tilt series images were collected at 0 and +/−68 degrees with 1 degree intervals. Images were aligned and reconstructed into tomogram volumes using Inspect3D software (FEI, Inc.) and rendered using Amira (Visage Imaging, Inc.).

## Results

### Cell Morphology and Virus Production of LGTV Infected Mammalian and Tick Cell Lines

**Figure 1:**
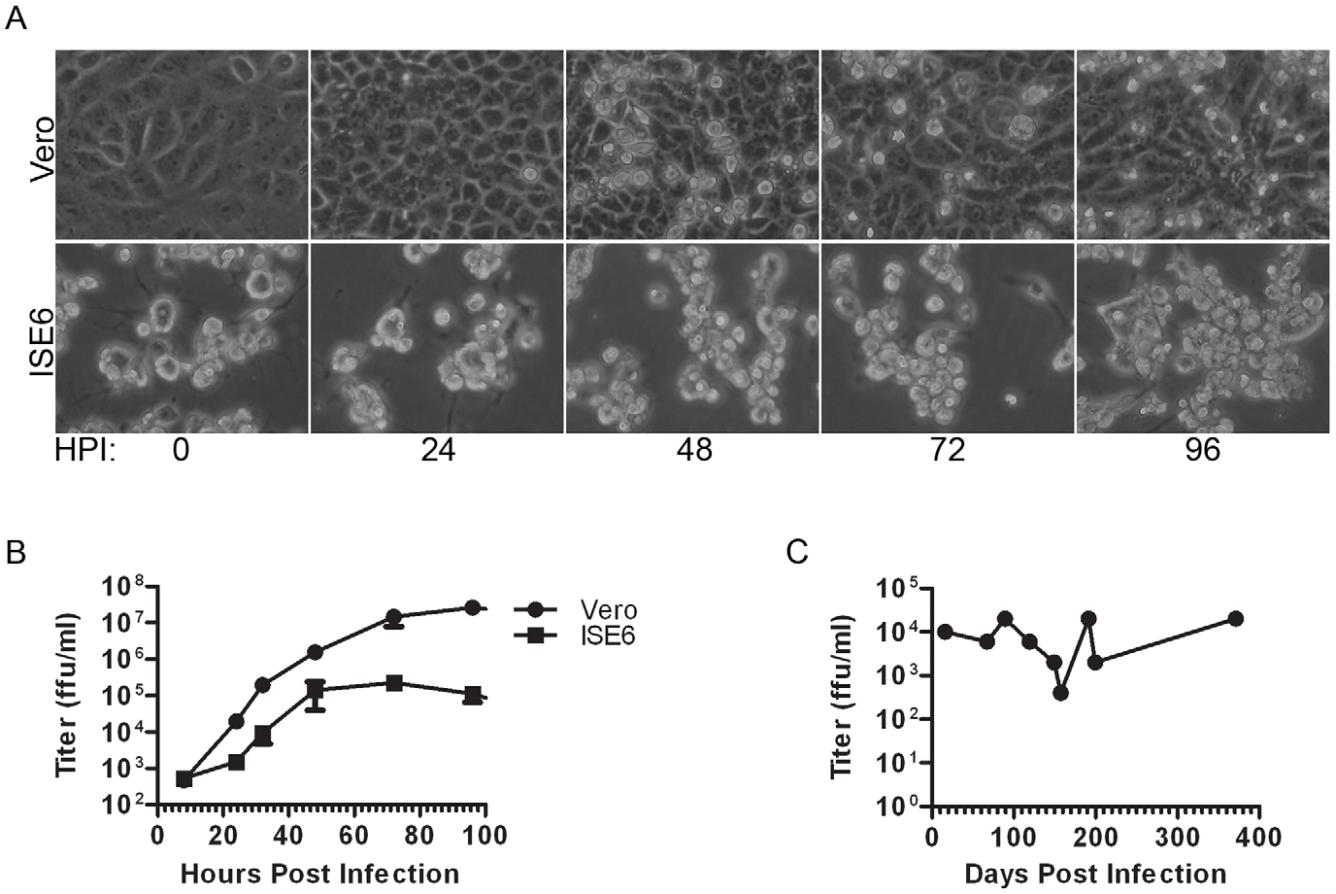
Replication of LGTV TP21 in cell culture. (A) Phase contrast images of Vero or ISE6 cells infected with LGTV TP21 (MOI 10) at indicated time points. (B) Supernatants from Vero or ISE6 cells at MOI of 10 were assayed for viral titer by immunofocus assay. Error bars indicate SEM from three independent experiments. (C) Supernatants from persistently infected ISE6 cells were assayed for viral titer by immunofocus assay.

Vector-borne flaviviruses replicate in both mammalian and arthropod cells. However, the three-dimensional (3D) fine structure of TBFV replication in mammalian cells has not yet been compared to that in acutely or persistently infected tick cells. In order to undertake this comparison, baseline characterizations of acutely infected mammalian (African green monkey kidney, Vero) and tick (*Ixodes scapularis* embryo derived, ISE6) cells were performed by light microscopy and virus titer was assayed from culture supernatants. Uninfected Vero cell cultures formed a monolayer of uniform elongated, fibroblast-like cells that were firmly adherent to the culture vessel. Infected Vero cells first showed cytopathic effect (CPE) by 24 hpi, consisting of rounded cells and syncytia (Fig. 1A). The CPE was progressive and by 96 hpi, Vero cells showed extensive evidence of cell death (shape irregularity, cytoplasmic condensation, blebbing, and an inability to exclude trypan blue). After 96 hours, very few cells remained attached to the culture vessel.

ISE6 cell cultures, on the other hand, are a mixed population of cells consisting of clumps of loosely adherent, round cells as well as more firmly adherent, stellate cells with branching pseudopodia. In marked contrast to infected Vero cell cultures, there was no obvious evidence of CPE following acute infection of the ISE6 cells (Fig. 1A). LGTV replicated well in both cell lines, but the titer was 2–3 logs lower in acutely infected ISE6 cells than in Vero cells (Fig. 1B). Thus, there was a prominent difference in the cellular response to acute LGTV infection between mammalian and tick cells. For subsequent experiments, we selected a time point of 48 hpi as a compromise between virus production and integrity of cellular morphology.

In nature, TBFV establish and maintain a persistent infection across the various life-stages of the arthropod host [8]. In an effort to model and study this aspect of TBFV infection *in vitro*, ISE6 cells were infected with LGTV, serially passaged at confluence, and examined at intervals for the induction and maintenance of a persistent infection. Interestingly, cultures of infected ISE6 cells appeared normal for a period of at least one year following initial infection. LGTV infection was maintained in the ISE6 cells, and the viral titer oscillated from a low of 2×10^3 ^ffu/ml to a high of 2×10^4^ ffu/ml (Fig. 1C). After roughly one year, approximately 50% of the cells were positive for viral proteins, and the culture supernatant at this point had a viral titer of 2×10^4^ ffu/ml (Fig. 1C). Thus, a persistent infection had been established in the ISE6 cells and that infection was still productive a year later.

### Distribution of LGTV Proteins in Infected Mammalian and Tick Cells

**Figure 2:**
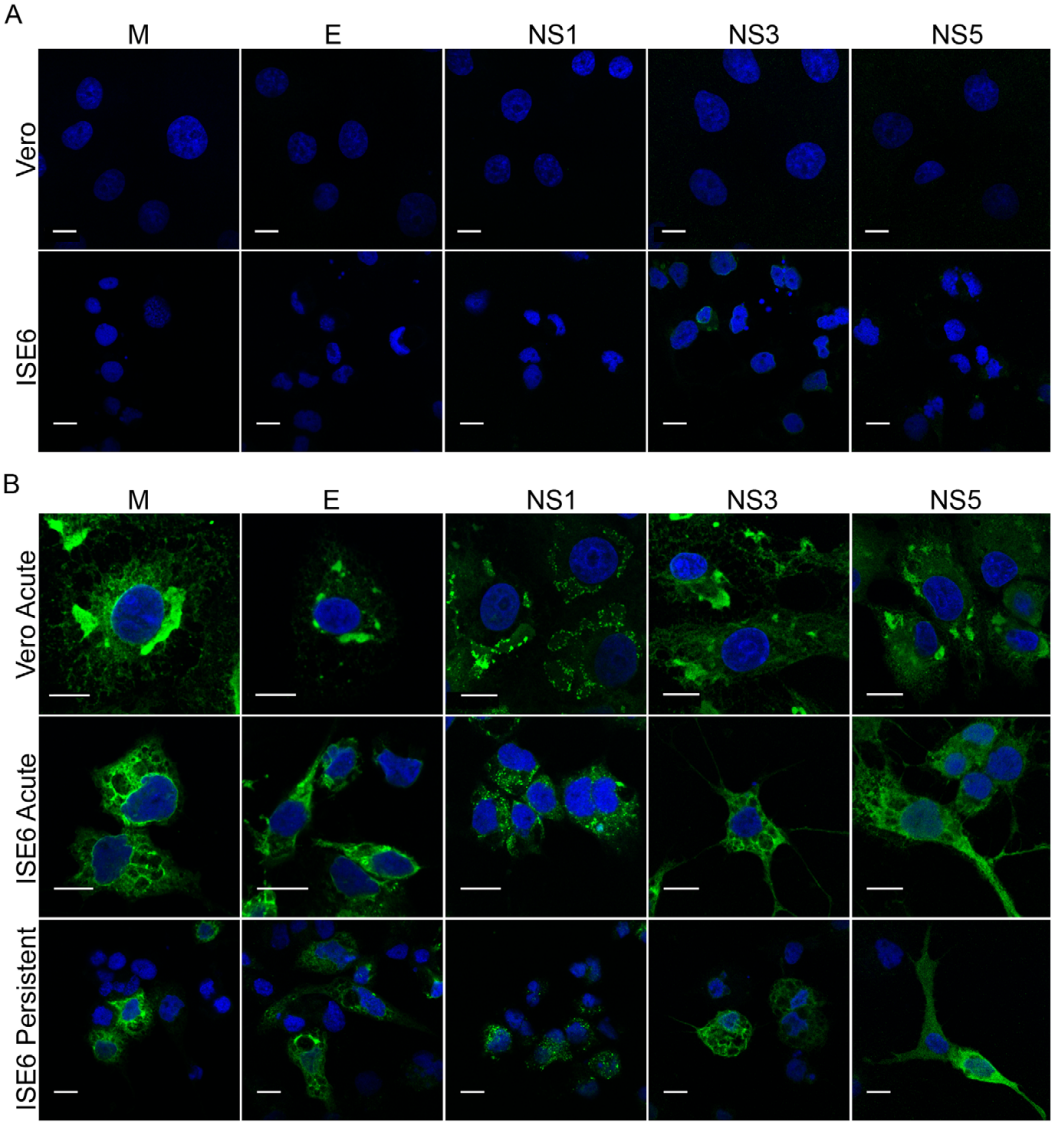
Distribution of viral protein labeling in Vero and ISE6 cells. Cells were infected at a MOI of 10, fixed, and stained for individual viral proteins. Nuclei were counterstained with DAPI (blue). Scale bars, 10 um (A) Mock-infected cells; (B) LGTV-infected cells.

The findings described in the previous section provided us with a model system in which to examine the distribution of several structural and non-structural proteins in infected mammalian and tick cells (Fig. 2). The analysis is complicated by the fact that the basic cellular morphology differs substantially between the mammalian and tick cell lines. Nevertheless, in acute infections in Vero and ISE6 cells, as well as in the persistently infected ISE6 cells, M, E, and NS3 proteins were localized to the perinuclear region, although some staining was seen throughout the cytoplasm (Fig. 2B). NS1 exhibited a more punctate cytoplasmic staining pattern and NS5 showed a diffuse cytoplasmic staining pattern with occasional evidence of nuclear staining. Similar patterns were seen for all the viral proteins in both cells lines.

**Figure 3:**
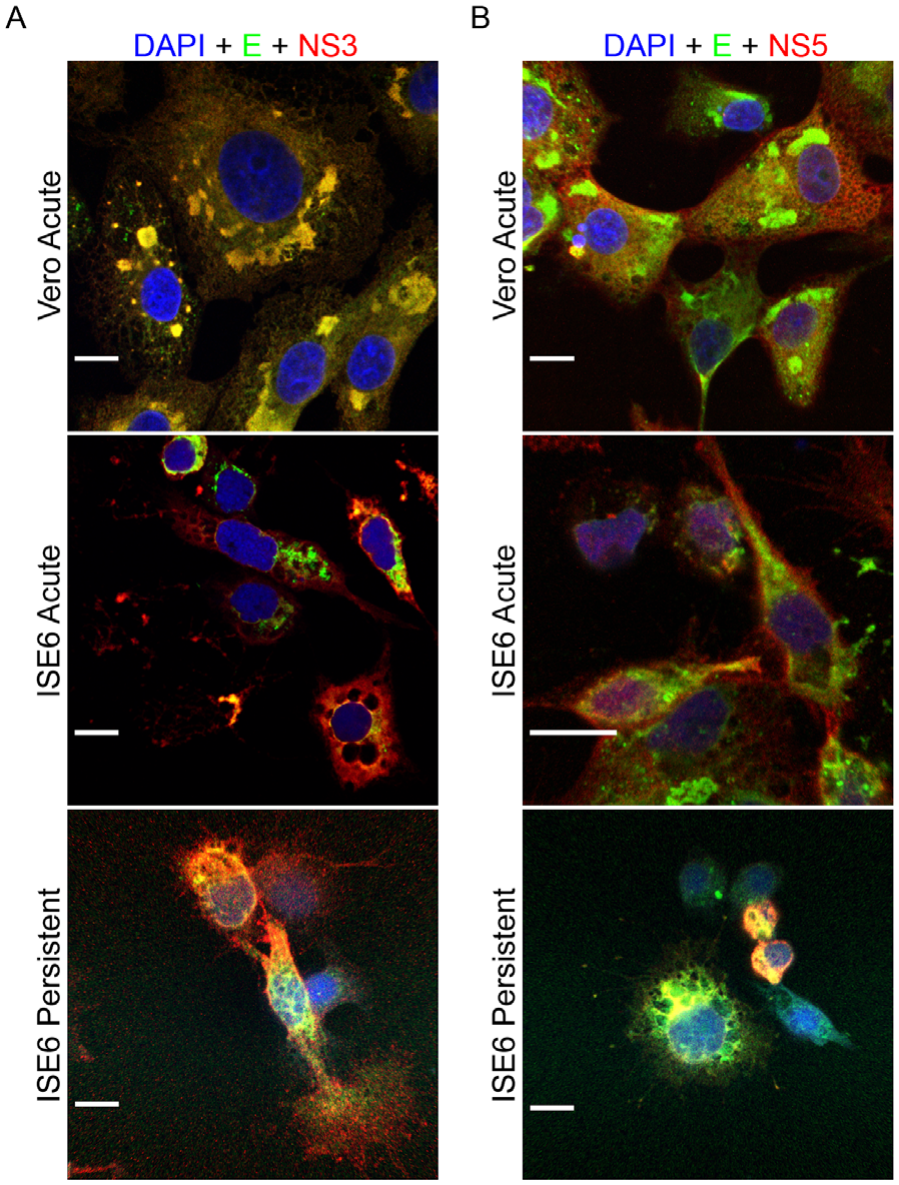
Colocalization of viral proteins in Vero and ISE6 cells. Cells were infected at a MOI of 10, fixed, and co-stained for the indicated viral proteins. Left panel (A): anti-E protein (green) and anti-NS3 protein (red), areas of colocalization between these two proteins appear yellow; Right panel (B): anti-E protein (green) and anti-NS5 protein (red), areas of colocalization between these two proteins appear yellow. Nuclei were counterstained with DAPI (blue). Scale bars, 10 um.

To more precisely evaluate the relative distribution of the proteins during infection, we co-stained specimens for multiple viral proteins. In each case, the bulk of NS3 staining was colocalized with both the viral membrane (prM) (data not shown) and envelope (E) proteins (Fig. 3A); although the overlap appeared less complete in the ISE6 cells. NS5 was diffusely distributed in the cytoplasm, but there was clear evidence that the staining for E overlapped (Fig. 3B). The punctate staining pattern for NS1 also exhibited coincidence with prM, E, and NS3 but with fewer areas of colocalization with NS5 (data not shown) in both Vero and ISE6 cells. There were no appreciable differences between the acute and persistently infected ISE6 cells (Fig. 3).

### Localization of LGTV Proteins with Markers for Cellular Organelles in Infected Mammalian and Tick Cells

**Figure 4:**
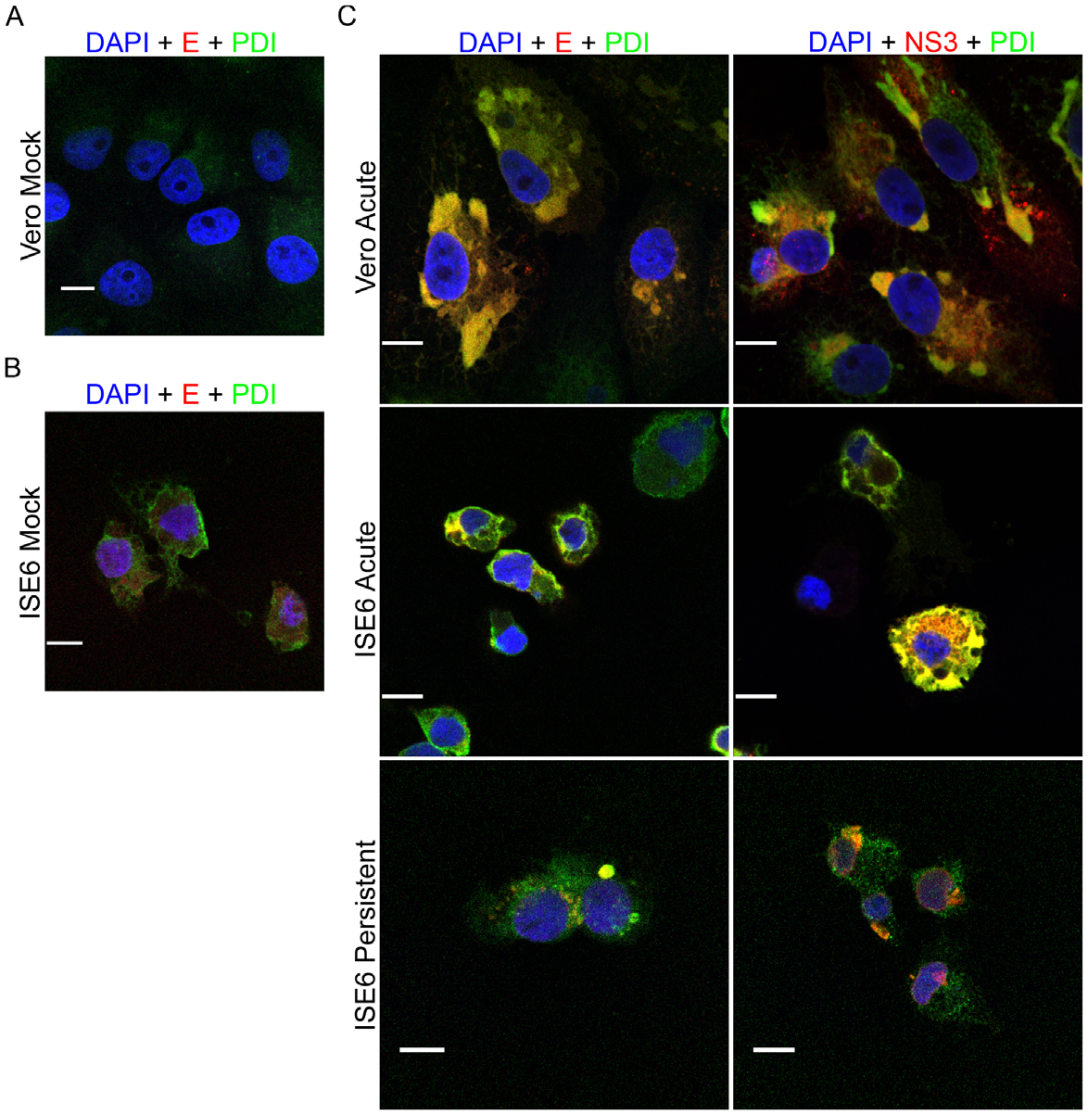
Majority of LGTV TP21 structural and nonstructural viral proteins localized to endoplasmic reticulum (PDI). Cells were infected at a MOI of 10, fixed, and co-stained with viral markers, anti-E or anti-NS3 (red), and anti-protein disulfide isomerase (PDI, green) to identify areas of endoplasmic reticulum. Areas of colocalization appear yellow. Nuclei were counterstained with DAPI (blue). Scale bars, 10 um. Mock-infected Vero (A) and ISE6 (B) cells are shown in left panels. Right panels (C) show images taken from infected cell cultures.

Flavivirus replication and assembly takes place on membranes derived from ER and that maturation of virus particles occurs within the trans-Golgi [40]–[43]. Thus, we next looked at LGTV protein localization in relationship to markers for ER (Dylight 488-conjugated protein disulfide isomerase, PDI) or Golgi membranes (Alexa Fluor 594-conjugated wheat germ agglutinin, WGA). In both infected mammalian and tick cells (Fig. 4C), we observed a notable increase in the amount of ER staining in comparison to mock infected cells (Fig. 4A & B), while the amount of Golgi staining remained relatively constant (date not shown). In infected Vero cells, the bulk of structural protein E and the nonstructural protein NS3 colocalized in large concentrated areas of ER (Fig. 4C) but a small amount of signal from E overlapped with the Golgi marker (WGA) (data not shown). Similar results were observed for both the acutely and persistently infected ISE6 cell cultures (Fig. 4C). Thus, LGTV infection of mammalian and tick cells was accompanied by an increase in ER related structures, and furthermore, both structural and nonstructural LGTV proteins are concentrated in those areas.

### Localization of dsRNA with Replicative Intermediates and Cellular Markers in Infected Mammalian and Tick Cells

**Figure 5:**
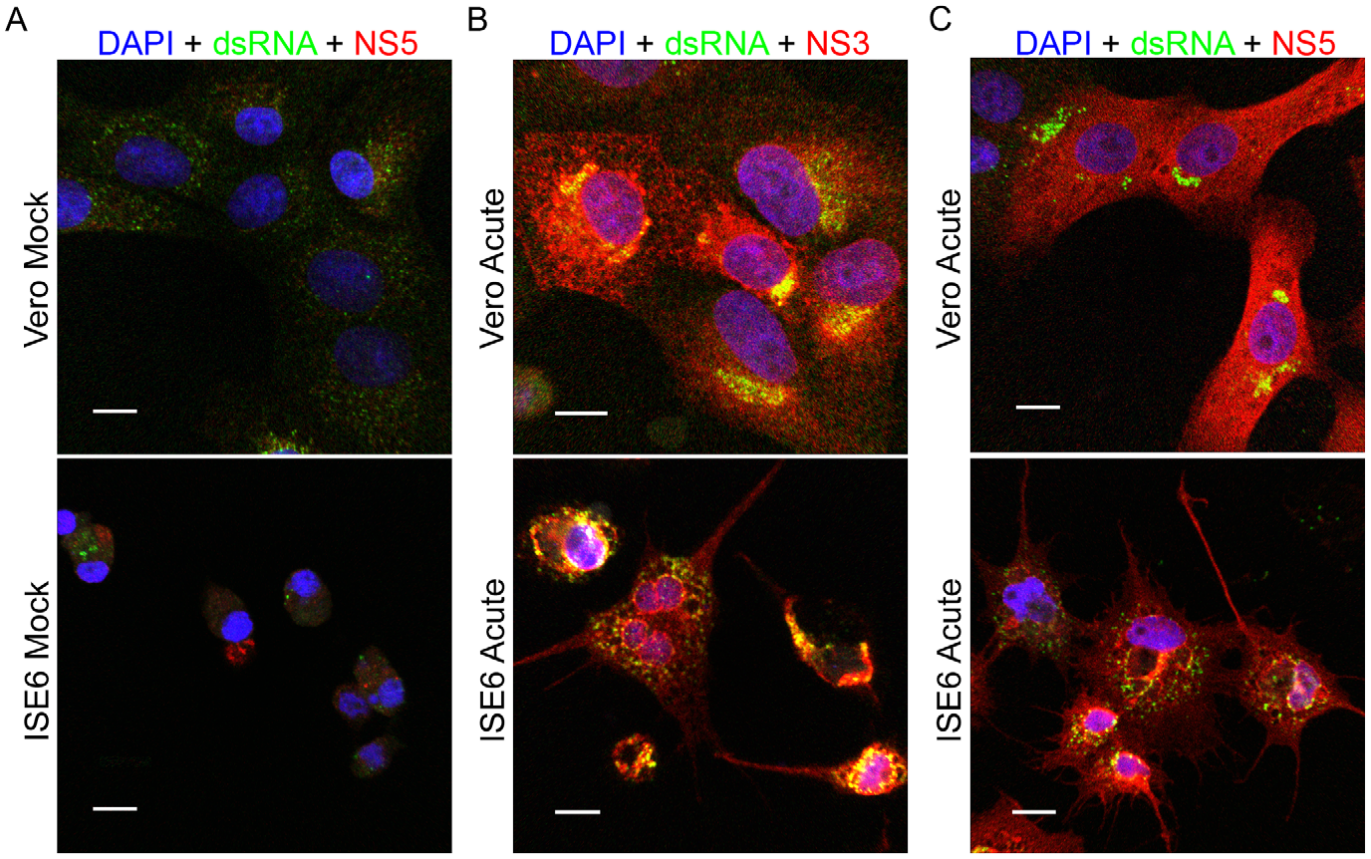
Colocalization of dsRNA with viral proteins in Vero and ISE6 cells. Cells were infected at a MOI of 10 and fixed at 48 hpi. Left panel (A): mock-infected cells were co-stained with anti-dsRNA (green) and anti-NS5 protein (red). Center panel (B): LGTV-infected cells were co-stained with anti-dsRNA (green) and anti-NS3 protein (red). Right panel (C): LGTV-infected cells were co-stained with anti-dsRNA (green) and anti-NS5 protein (red). Nuclei were counterstained with DAPI (blue). Scale bars, 10 um.

**Figure 6:**
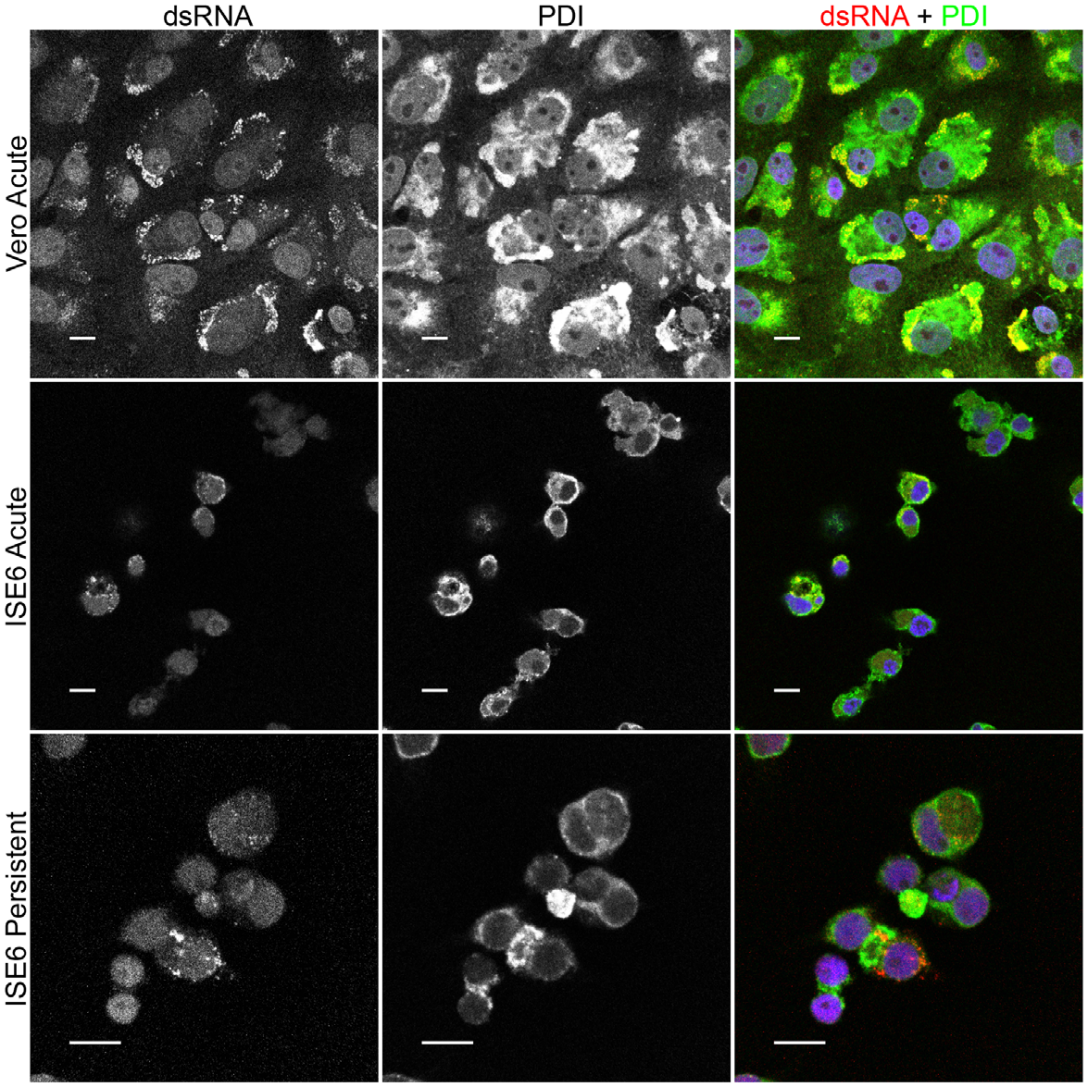
Localization of LGTV TP21 replication complex to endoplasmic reticulum. Cells were infected at a MOI of 10, fixed, and co-stained for double-stranded RNA (dsRNA, red) and endoplasmic reticulum (protein disulfide isomerase (PDI), green). Nuclei counterstaining (DAPI, blue) is only shown in the merge panel. Scale bars, 10 um.

An obligate marker for flavivirus replication is the presence of the replicative form, double-stranded RNA (dsRNA). Therefore, to further demonstrate that LGTV replication was occurring in an ER derived compartment [44], we co-stained infected Vero and ISE6 cells for dsRNA and either viral proteins or cellular markers. Uninfected cells showed no dsRNA staining (Fig. 5A). In infected Vero cells, labeling for dsRNA was largely confined to areas staining for NS3 (Fig. 5B) and NS5 (Fig. 5C). The same general pattern was seen in acutely (Fig. 5B and 5C) and persistently (data not shown) infected ISE6 cells, but the dsRNA signal was not as prominent as in the Vero cells. The majority of dsRNA labeling was coincident with ER staining (Fig. 6), but not with Golgi staining (data not shown) in acutely infected Vero or in ISE6 cells, both acutely and persistently infected. Taken together, these findings indicated that in both mammalian and tick cells, LGTV replication, as defined by the presence of dsRNA, viral helicase, and viral polymerase, was occurring in areas of ER proliferation.

### Ultrastructural Comparison of LGTV Infection in Mammalian and Tick Cells

**Figure 7:**
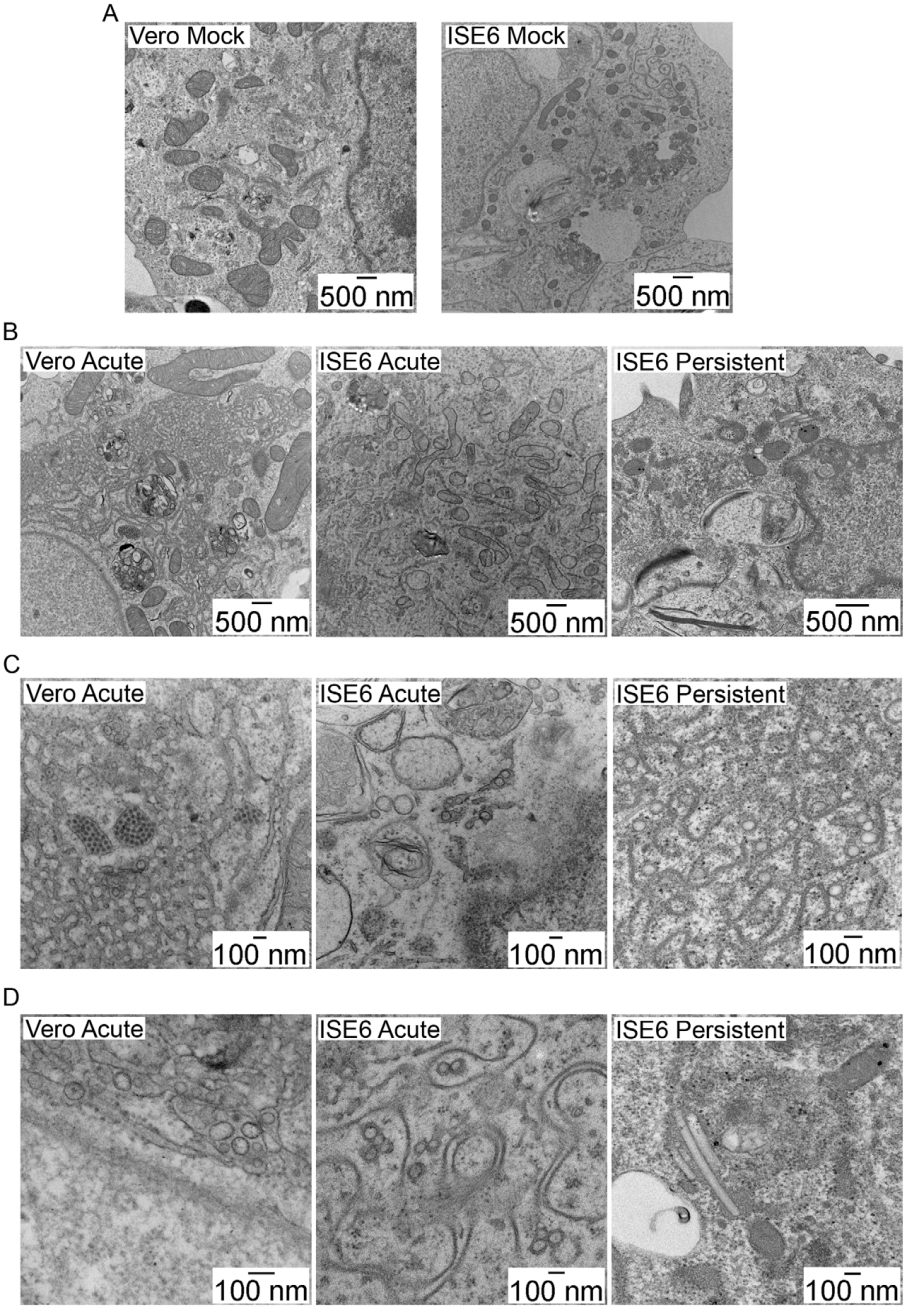
Ultrastructure of acute and persistent LGTV TP21 infection. Cells were infected at a MOI of 10, fixed, resin embedded, 70 nm section cut, and processed for transmission electron microscopy (see Materials and Methods). (A) Mock infected (B) Lower magnification showing ER proliferation and vesicles. (C) Higher magnification showing round vesicles surrounded by ER membrane. (D) Round vesicles, elongated vesicles, and tubules. Scale bars shown in inset.

In the previous sections, we showed that LGTV replication was occurring in part of the cytoplasm associated with expanded ER and that there was no obvious difference between mammalian and tick cells. However, immunofluorescence does not provide sufficient resolution to clearly define the cellular compartments associated with replication. Therefore, to compare TBFV infection in mammalian and tick cells at higher resolution, we used TEM of fixed, resin-embedded specimens to examine mock-infected Vero and ISE6 cells (Fig. 7A), LGTV infected Vero, and either acutely or persistently infected ISE6 cells.

In Vero cells, extensive areas of altered membrane proliferation (Fig. 7B–D) could be found in the majority of cells, consistent with the expansion of the ER system evident by fluorescence microscopy [27], [29], [30]. In some sections, these regions of proliferation encompassed nearly half of the cytoplasmic area, thus explaining our immunofluorescence results. Single-membrane bound vesicles, ranging in diameter from 60–100 nm, were frequently found within these proliferated ER areas, often occurring in large groups contained within dilated ER cisternae. Single, small groups, and large paracrystalline arrays of virions were readily observed (Fig. 7A). Infrequently, tubular structures or elongated vesicles were seen (Fig. 7D). Tubules have been seen in other reports on ultrastructure of mosquito-borne flavivirus mammalian cell infection [30], [45] and mosquito cell infection [26], [46], [47]; however, no function has been assigned to these tubules.

In acutely infected ISE6 cells, membrane proliferation and vesicles were also observed (Fig. 7B & C), but the extent was notably less widespread than in mammalian cells, consistent with the findings of previously published work [35]. The vesicles were also found in dilated ER cisternae and were the same diameter as those seen in Vero cells (Fig. 7C). Virions were not detected until 96 hpi (data not shown), likely a consequence of the lower viral titer and slower replication observed in the tick cells (Fig. 1B). The membranous tubules or vesicles were present at a slightly higher frequency than in acutely infected Vero cells.

In persistently infected ISE6 cells, membrane proliferation was more extensive than in acutely infected tick cells; but, again, this was not as pronounced as in infected Vero cells (Fig. 7B). Vesicles were again seen within proliferated, dilated ER and remained within the same size range as in acute infection. Virions were not noted in the samples we examined. One difference in the persistently infected ISE6 cells was particularly striking. Tubules were seen in almost all persistently infected ISE6 cells and often occurred in fascicle-like bundles of multiple tubules cloaked by a single membranous sheath (Fig. 7D).

### Three-dimensional Evaluation of LGTV Infection in Mammalian and Tick Cells

**Figure 8:**
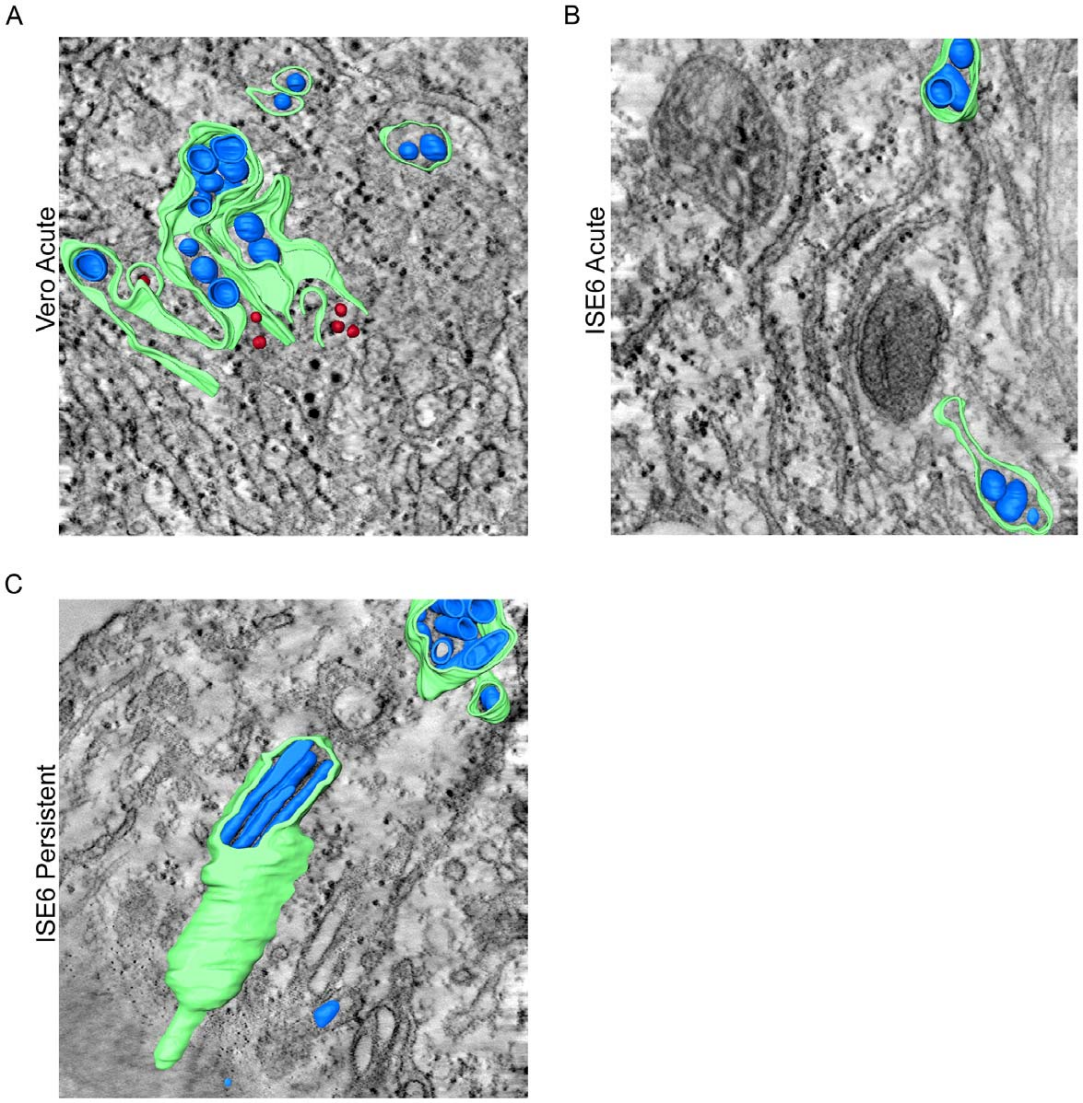
Electron tomography of LGTV TP21 infected Vero and ISE6 cells. Cells were infected at a MOI of 10, fixed, epoxy resin embedded, and 250 nm sections cut for dual-axis electron tomography. Panels show the 3-D surface rendering of convoluted membrane (green), vesicles (blue) and virions (red) derived from the 3-D reconstruction. (A) Vero Acute, (B) ISE6 Acute, and (C) ISE6 Persistent.

**Figure 9:**
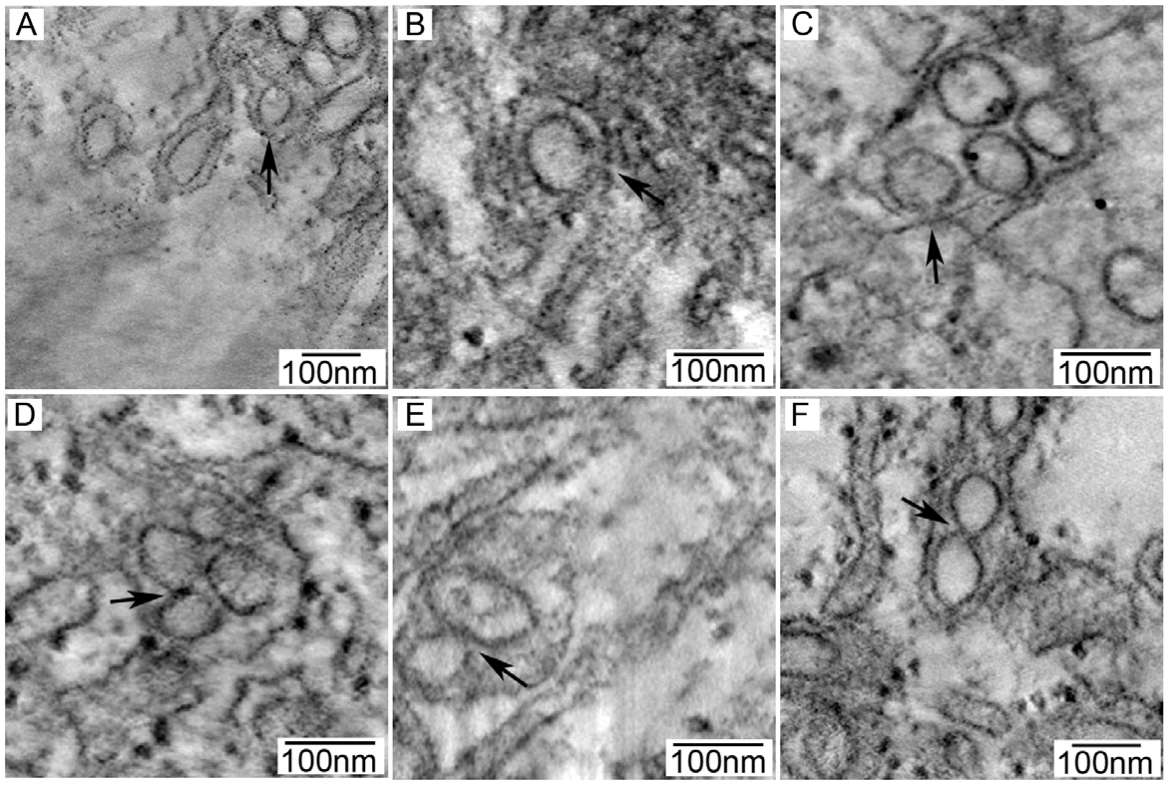
Visualization of vesicles open to cytoplasm or interconnected to each other in LGTV TP21 infected Vero and ISE6 cells by ET. Cells were infected at a MOI of 10, fixed, epoxy resin embedded, and 250 nm sections cut for dual-axis electron tomography. All panels are cropped areas of a single slice from the 3-D reconstruction. Vesicles within areas of ER proliferation showed connections (marked with arrows) that were either vesicle to cytoplasm or vesicle to vesicle. (A–C) Vesicle to cytoplasm openings, (D–F) Vesicle to vesicle connections. (A & D) Acutely infected Vero cells; (B & E) Acutely infected ISE6 cells; (C & F) Persistently infected ISE6 cells.

The recent application of electron tomography (ET) to the study of virus infection has allowed 3D evaluation of virus replication [48]–[53] including in mosquito-borne flavivirus infection [30], [31]. We employed ET of 250 nm thick, fixed, and resin-embedded specimens to enhance the findings described in the earlier sections. Tilt series were acquired, aligned using gold particles, and tomograms assembled to reveal 3D structures. In Vero cells acutely infected with LGTV, we found the vesicles and virions were contained within an anastomosing network of dilated membranes. The 60–100 nm diameter vesicles were round in shape (Fig. 8A and Movie S1) and there were pore-like openings connecting vesicles to the cytoplasm (Fig. 9A) and to other vesicles (Fig. 9D).

In tick cells, acute LGTV infection induced 3D rearrangements similar to those seen in Vero cells (Fig. 8B and Movie S2). The membrane proliferation, while not as marked as in Vero cells, still resulted in a continuous network that surrounded the vesicles. The majority of vesicles observed in the tick cells were round (Fig. 7C & D); however, slightly elongated vesicles were seen more frequently than in Vero cells. Communicating pores or openings between the vesicles and the cytoplasm (Fig. 9B) and between individual vesicles (Fig. 9E) were also seen in the tick cells.

The persistently infected tick cells also contained round vesicles enclosed within dilated membrane structures (Fig. 8C and Movie S3). The 3D analysis confirmed that the elongated profiles were in fact tubular, and that they were clustered in fascicle-like groups surrounded by a single membrane. These structures were cylindrical in shape with closed ends, varying in width from 60 to 100 nm and in length from 100 to 800 nm. Pores were seen between tubules and vesicles (Fig. 9C & F); however, in our examinations of numerous tubular structures, we were unable to identify pores linking tubules with other tubules or with the cytoplasm.

## Discussion

Studies of flavivirus infected cells have yielded significant insights and important information about virus reproduction and the consequences of infection on host cell biology and cellular structure. Similar to other positive strand viruses, such as Semliki Forest virus [54], rubella virus [52], and poliovirus [51], the flaviviruses induce remarkable alterations in the cytoplasmic membrane system. In our study, Vero cells acutely infected with LGTV show the ER proliferation, vesicle accumulation, and membrane network (Fig. 7B–D) described in earlier studies with mosquito-borne flaviviruses [30], [31]. A number of functions have been ascribed to the alterations, including concentration of viral replication machinery, provision of a solid state platform for viral protein synthesis and replication, and sequestration of viral dsRNA replicative form from innate immune sensors [27], [55], [56]. In general, viral protein distribution in acutely infected tick cells was similar; however, some differences were apparent. Vero cells had higher levels of ER proliferation and rearrangement as well as significantly more viral particles. This may have been the result of the greater level of replication seen in Vero cells but, it is also possible that the differences are the result of a fundamental difference between the two host cell lines.

In our study, an absence of observed viral particles in LGTV-infected ISE6 cells did not allow for confirmation of previously reported differences seen with TBEV [35] between virus particle location in mammalian versus arthropod cells. However, we have augmented this earlier published work by reporting the 3D structure of acutely infected tick cells. By ET, the proliferating membranes are revealed to be a complex anastomosing system of membranes almost certainly derived from ER. In the Semliki Forest virus replicon system containing Kunjin virus proteins, expression of a single flavivirus protein, NS4A, can induce the membrane rearrangements [22], and similar results have been reported for Dengue virus NS4A [57], but it is uncertain at this time if this role can be assigned to NS4A in the TBFV. Future investigation is in progress to determine which TBFV protein or combination of proteins cause membrane rearrangement in mammalian and tick cells.

The circular profiles seen in TEM were clearly demonstrated by ET to be spherical vesicles bound by a single membrane and to have pore-like connections to the cytoplasm or to other vesicles in Vero and ISE6 cells. The function of the vesicles is thought to be to minimize exposure of the replicative form dsRNA to innate immune sensors, such as RIG I [27], [58], [59], while the pores are thought to provide a conduit for nucleotides, amino acids, and other components required for replication and gene expression [30]. In brief, our findings suggest marked similarities between acute replication in ISE6 and Vero cells, and further demonstrate the similarity between cellular features of acute tick-borne and mosquito-borne [30], [31] flavivirus replication.

Our work also allowed a detailed examination of cellular features of persistent LGTV infection in ISE6 cells and an opportunity to compare acute and persistent infection in tick cells. Over a yearlong period, the persistently infected ISE6 cells maintained a grossly normal morphology. Furthermore, the distribution of viral proteins and their co-localization with cellular markers mirrored the acute infection.

However, study by EM and 3D reconstruction revealed differences between the persistently infected and the acutely infected ISE6 cells. The persistently infected tick cells had greater changes in ER structure and ER abundance (Fig. 7B–D) than the acutely infected cells, although viral titers (Fig. 1B & C) were similar in both settings. However, the most remarkable difference between acute and persistent infection was the number of tubular structures. These structures have been noted infrequently in infected cells [26], [30], [34], and their relevance in flavivirus replication is uncertain. In our acute mammalian and tick cell experiments, we confirmed these observations and saw tubules only occasionally (Fig. 7D, 8, and Movies S1, S2, and S3). However, in the persistently infected tick cells, the number of tubules increased dramatically (Fig. 7D, 8C, & Movie S3). While the diameter was similar to that seen in round vesicles (60–100 nm), the length was up to eight times as long. The tubules were often arranged in membrane-bound, fascicle-like bundles (Fig. 8C and Movie S3). The 3D reconstructions demonstrated that the tubules were closed on each end and, although closely juxtaposed, were not connected by pores to other tubules or to the cytoplasm, unlike the round vesicles. The function of the tubules is obscure at this time, and it is unclear if they represent bona fide features of replication, aberrant structures, or the result of a cellular process to restrict infection. It is possible the tubules may play a role in initiation or maintenance of the persistent infections or if the apparent lack of pores in the tubules is consequential. It may be that the increase in the number of tubules is the result of the higher number of defective virus particles, which are known to exist in persistently infected cells [60]–[62]. Tubules may be formed as a result of a failure to correctly gather the membrane to form the round vesicles and pores that are associated with the flavivirus replication complex. Perhaps the lack of pores prevents proper replication or packaging of the viral genome. Additional cell biology or biochemistry studies may shed light on the role of the tubules.

In summary, our experiments have provided the first analysis of the 3D structure of tick-borne flavivirus infection in both mammalian and arthropod host cell systems. We observed vesicles with pores connecting to other vesicles or opening to the cytosol in tick-borne flavivirus infection, similar to those seen in mosquito-borne flavivirus infection. We have shown for the first time the 3D ultrastructure of acutely and persistently, flavivirus-infected arthropod cells, facilitating the observation of the shift that occurs from round vesicles during acute TBFV-infection to the elongated tubules that dominate persistent infection. Future experiments are needed to better understand the increasing presence of tubules at the site of membrane rearrangement during persistent infection.

## Supporting Information

Movie S1 **LGTV TP21-induced structures in acutely infected Vero cells.** Animation through a z-series and 3D surface rendering of a semi-thick section of an acutely infected Vero cell. ER is depicted in green, vesicles in blue, and virions in red. Both virions and vesicles are contained within a network of proliferated ER. The images were aligned using Inspect3D software (FEI, Inc.) and rendered using Amira (Visage Imaging, Inc., San Diego, CA).
(MP4)

Movie S2 **LGTV TP21-induced structures in acutely infected ISE6 cells.** Animation through a z-series and 3D surface rendering of a semi-thick section of an acutely infected ISE6 cell. ER is depicted in green and vesicles & tubules in blue. Vesicles and tubules are contained within proliferated ER. Tubules are short in length, only reaching approximately twice the diameter length. The images were aligned using Inspect3D software (FEI, Inc.) and rendered using Amira (Visage Imaging, Inc., San Diego, CA).
(MP4)

Movie S3 **LGTV TP21-induced structures in persistently infected ISE6 cells.** Animation through a z-series and 3D surface rendering of a semi-thick section of a persistently infected ISE6 cell. ER is depicted in green and vesicles & tubules in blue. Numerous long tubules are seen in a large bundle; however, smaller tubules and round vesicles are also seen. The images were aligned using Inspect3D software (FEI, Inc.) and rendered using Amira (Visage Imaging, Inc., San Diego, CA).
(MP4)
